# Binary Oxides Prepared by Microwave-Assisted Solution Combustion: Synthesis, Characterization and Catalytic Activity

**DOI:** 10.3390/ma12060910

**Published:** 2019-03-19

**Authors:** Kawthar Frikha, Lionel Limousy, Jamel Bouaziz, Kamel Chaari, Ludovic Josien, Habiba Nouali, Laure Michelin, Loic Vidal, Samar Hajjar-Garreau, Simona Bennici

**Affiliations:** 1 Université de Haute-alsace, CNRS, IS2M UMR 7361, F-68100 Mulhouse, France; kawthar.frikha@uha.fr (K.F.); lionel.limousy@uha.fr (L.L.); ludovic.josien@uha.fr (L.J.); habiba.nouali@uha.fr (H.N.); laure.michelin@uha.fr (L.M.); loic.vidal@uha.fr (L.V.); samar.hajjar@uha.fr (S.H.-G.); 2 Université de Strasbourg, F-67000 Strasbourg, France; 3 Laboratoire de Chimie Industrielle, Ecole Nationale d’Ingénieurs de Sfax, Université de Sfax, Sfax BP1173, Tunisie; jamel.bouaziz@enis.rnu.tn (J.B.); kamel.chaari@enis.rnu.tn (K.C.)

**Keywords:** alumina-based Ni, Cu, Co-oxide catalysts, microwave-assisted solution combustion, CO oxidation, catalytic activity

## Abstract

Three different alumina-based Ni, Cu, Co oxide catalysts with metal loading of 10 wt %, and labeled 10Ni–Al, 10Co–Al and 10Cu–Al, were prepared by microwave-assisted solution combustion. Their morphological, structural and surface properties were deeply investigated by complementary physico-chemical techniques. Finally, the three materials were tested in CO oxidation used as test reaction for comparing their catalytic performance. The 10Cu–Al catalyst was constituted of copper oxide phase, while the 10Ni–Al and 10Co–Al catalysts showed the presence of “spinels” phases on the surface. The well-crystallized copper oxide phase in the 10Cu–Al catalyst, obtained by microwave synthesis, allowed for obtaining very high catalytic activity. With a CO conversion of 100% at 225 °C, the copper containing catalyst showed a much higher activity than that usually measured for catalytic materials of similar composition, thus representing a promising alternative for oxidation processes.

## 1. Introduction

Different synthetic methods, such as co-precipitation, impregnation, sol-gel and homogeneous deposition precipitation have been widely used for catalytic materials preparation. These methods generally require numerous synthetic steps, specific equipment and energy intensive protocols, which pushed researchers to look for alternative methods. Solution combustion is a novel method for catalyst preparation [1,2,3,4,5]. This synthetic method typically involves an exothermic redox reaction and does not require any special equipment and process control. The properties of the materials obtained by solution combustion strongly depend on the processing parameters and can be tuned by varying the combustion conditions. The success of this process is then closely linked to the choice of the redox mixture and the most suitable precursors. The stoichiometry calculation is based on the principles used in propellant chemistry and consists of balancing the elemental oxidizing and reducing valences of the redox compounds [6,7]. Salts like nitrates, metal sulfates and carbonates are chosen as oxidizing agents, and fuels such as glycine, citric acid and urea are widely used as reducing agents. A suitable fuel must be water soluble, generated a large amount of harmless gases, and should present a low ignition temperature [7]. Metal nitrates are chosen as metal precursors due to their high water solubility, allowing a greater homogenization. Urea is one of the most suitable fuels because of its high solubility in water, the compatibility with metal nitrates, and the possibility to carry on the combustion reaction in a more controllable way [8]. The reaction is initiated by the fast heating of the aqueous solution up to the ignition temperature, followed by the spontaneous combustion of the mixed solution. The heat required for initiating the combustion is often supplied by means of an electrical heating such as muffle ovens [9,10], hot plate [11,12] and heating mantle [13]. These traditional heating processes lead to a temperature gradient between the interior and exterior of the solution, resulting in a regional inhomogeneity of the prepared material. Recent research proposed an alternative method for the combustion initiation by microwave heating. Microwave irradiation presents several advantages in comparison with more conventional methods; it shows short reaction time, small particle sizes, narrow size distributions and high purity of the obtained materials [14]. Under microwave irradiation, the solution mixture is uniformly heated, allowing the formation of more uniform materials that exhibit superior catalytic activity [15,16].

In the present paper, microwave assisted solution combustion has been chosen in order to prepare more effective catalysts for the total oxidation of carbon monoxide. This test reaction was chosen in relation to the fact that air pollution is one of the most serious environmental problems, and carbon monoxide is often one of the components of atmospheric pollution, being largely produced from vehicle emissions and industrial activities. Over the last fifty years, many important advances in catalytic research have introduced a variety of solutions to air pollution control and efficient heterogeneous catalysts have been developed for environment-related applications [17,18,19,20,21]. In automotive exhaust emission control, the catalytic oxidation of carbon monoxide (2CO + O_2_ → 2CO_2_) is very important to meet the increasingly stringent environmental regulations in an effective way [22]. Furthermore, since the classic studies of Langmuir [23], CO oxidation has often been considered as an ideal test reaction for fundamental investigations in heterogeneous catalysis. CO oxidation on supported noble metal catalysts has gained significant interest over the past half century. The high cost and low availability of noble metals pushed to substitute precious metals by less costly transition metal oxide catalysts [24,25,26]. The most reactive metal oxide catalysts towards oxidation–reduction reactions are those presenting p-type semiconducting properties, as cobalt, nickel and copper oxides. Their enhanced activity is due to the insertion of excess oxygen in their lattice with subsequent creation of cationic vacancies [27]. Furthermore, among the various metal oxide catalyst supports, alumina remains the most used due to its mechanical and physico-chemical properties. For example, CO oxidation over cobalt, copper or nickel oxide-based catalysts supported on alumina has been investigated by various research groups [28,29,30,31,32,33,34,35]. Yao et al. [28] compared two methods for preparing Co_3_O_4_/Al_2_O_3_ catalysts; mechanical mixing and impregnation process. The catalyst obtained by mechanical mixing showed a higher activity than that prepared by impregnation. Indeed, the impregnation of acidic cobalt salts on alumina can induce a strong interaction, with the formation of cobalt aluminate, and a decrease in activity. A similar experimental method was applied to prepare another type of catalyst, constituted of cobalt oxide deposited on alumina as support [29], which showed high CO conversion at low temperature in a continuous-flow reactor. Wang et al. [30,31] compared the behavior of Co_3_O_4_-based catalysts supported on SiO_2_, Al_2_O_3_ and TiO_2_. They found that, at 700 °C, alumina and titania gave rise to strong interactions with Co_3_O_4_, with formation of cobalt aluminate (CoAl_2_O_4_) or cobalt titanate (CoTiO_3_), which are inactive phases in CO oxidation. The oxidation of CO over copper oxide has been studied since 1923. Yao et al. [32] compared the behavior of CuO and Co_3_O_4_ mixed with (or impregnated on) alumina or zirconia in CO oxidation. They found that the catalyst containing γ-Al_2_O_3_ was less active (per unit of surface area) than that containing ZrO_2_ or α-Al_2_O_3_. The low activity of the Co_3_O_4_ or CuO catalysts supported on ɣ-Al_2_O_3_ has been attributed to the formation of two spinels: CoAl_2_O_4_ and CuAl_2_O_4_. In 1967, Pierron et al. [33] investigated by in situ X-ray powder Diffraction (XRD) the structure of the copper species in a CuO/Al_2_O_3_ catalyst and their correlation to the catalytic activity in CO oxidation. Their results confirm that the presence of Cu_2_O is required for a good activity. Luo et al. [34] studied the process of solid–solid interaction between CuO and Al_2_O_3_ and its effect on the CO oxidation activity. The authors assumed that the formation of CuAl_2_O_4_ leads to a decrease in the CO oxidation activity. On the other hand, the oxidation of CO over nickel oxide-based catalysts was much less widely reported; indeed, nickel oxide does not exhibit good performances in CO oxidation, when compared to cobalt and copper oxides [25]. Chen et al. [35] tested the catalytic activity of NiO deposited on Al_2_O_3,_ which exhibited a CO conversion of only 50% at 625 °C.

The aim of this study was then to prepare improved alumina-based Ni, Cu, Co-oxide catalysts by microwave-assisted solution combustion. The impact of the structural, morphological and surface properties of the catalyst was then evaluated on their catalytic activity in CO oxidation. To the best of our knowledge, no similar investigations on CO oxidation over Ni–Al, Cu–Al, Co–Al binary oxide catalysts prepared by microwave-assisted solution combustion method have been reported.

## 2. Materials and Methods

### 2.1. Materials Preparation

The starting materials used in this work were: aluminum nitrate nonahydrate (Al(NO_3_)_3_·9H_2_O, ≥98% Fluka, Steinheim am Albuch, Baden-Württemberg, Germany), urea (CO(NH_2_)_2_, >99% Fluka), copper nitrate trihydrate (Cu(NO_3_)_2_·3H_2_O, ≥99.5% Merck, Darmstadt, Hessen, Germany), nickel nitrate hexahydrate (Ni(NO_3_)_2_·6H_2_O, ≥99% Merck), and cobalt nitrate hexahydrate (Co(NO_3_)_2_·6H_2_O, ≥99% Merck). In all cases, the precursors’ proportions in the initial mixture were calculated to maintain the molar ratio of reducing valences from urea to oxidizing valences from metal nitrates (RV/OV) equal to 1.1, according to Jain et al.’s propellant theory [6]. The choice of the RV/OV molar ratio was made to produce a high flame temperature, resulting in the formation of highly pure and homogeneous powders. Ni, Co and Cu precursors were added in order to obtain 10 wt % metal content in the 10Ni–Al, 10Co–Al and 10Cu–Al catalysts.

At first, appropriate amounts of aluminum nitrate and metal nitrate M(NO_3_)_2_·xH_2_O (where M = Ni, Cu or Co) were dissolved in the minimum volume of doubled distilled water and magnetically stirred until complete dissolution. Then, the appropriate amount of urea was added into the solution under continuous stirring, during about 1 h at 60 °C, to evaporate the excess of water. The solution was then transferred into a Pyrex beaker and heated in a microwave oven working at 700 W, 2.45 GHz. During a typical microwave heating process, the aqueous solution starts to boil and eventually decomposes. Then, a spontaneous ignition starts up the flaming type combustion, accompanied to the releasing of large quantities of gases, takes part. Foamy and low-density powders are then produced. The entire combustion process ends-up in less than 5 min. After synthesis, the obtained powders were calcined in a muffle oven at 500 °C for 12 h.

For comparison purposes, an alumina sample was prepared through microwave-assisted solution combustion, under identical experimental conditions, using aluminum nitrate-urea mixture. The obtained powder was then calcined in a muffle oven at 500 °C for 2 h.

### 2.2. Materials Characterization

The catalysts metal loading (wt %) was determined by Wavelength Dispersive X-ray Fluorescence (WDXRF) in a Philips MagiX (Eindhoven, The Netherlands) (2.4 kW) spectrometer. Previous to analysis, pellets of the samples (13 mm of diameter and 1 mm of thickness) were obtained by pressing the powders under 7-ton pressure during 5 min. 

X-ray Diffraction (XRD) analysis was carried out using a PANalytical MPD X’Pert Pro diffractometer (Eindhoven, The Netherlands) operating with Cu Kα radiation, λ = 0.15406 nm at 40 mA and 45 kV. Data were recorded at room temperature, within the 2 theta (°2θ) range of 10–90°, in step size of 0,017° and scan step time of 220 s. XRD patterns were indexed according to the Joint Committee on Powder Diffraction Standards (JCPDS) database cards provided by the International Center for Diffraction Data ICDD.

The textural properties of calcined catalysts and calcined alumina were obtained from the nitrogen adsorption/desorption isotherms acquired at −196 °C with a Micromeritics ASAP 2040 (Norcross, GA, USA) apparatus. Prior to the measurements, the powders were degassed under vacuum at 200 °C for 10 h to remove the physisorbed gases. The specific surface areas were calculated by the Brunauer–Emmett–Teller (BET) method. Total pore volume and average pore diameter were determined using the standard Barrett–Joyner–Halenda (BJH) method applied to the desorption branch of the isotherm.

X-ray photoelectron spectroscopy (XPS) measurements were done on a VG Scienta SES 2002 spectrometer (Uppsala, Sweden) equipped with a monochromatic Al Kα X-ray source (Al Kα = 1486.6 eV) and a hemispherical analyzer (Uppsala, Sweden). The spectra were recorded using a pass energy of 100 eV, along with pressure of 10^−9^ Pa in the analysis chamber. The analyzed zone has a surface of 24 mm^2^ and an analysis depth of 9 nm. Binding energies (BEs) were calibrated by taking C 1s peak (284.7 eV) of contaminating carbon as reference. The peaks were fitted by Gaussian-Lorentzian functions using the XPS-CASA software (casaXPS software 2.3.18 Ltd., Teignmouth, UK).

Scanning electron microscopy (SEM) was carried out in a Philips XL30 microscope at an accelerating voltage of 7 kV. Prior to the observations, the powders were spread onto the surface of a carbon tape and sputter-coated with carbon.

Transmission electron microscopy (TEM) was performed on a Jeol ARM-200F microscope (Tokyo, Japan), operating at 200 kV. Prior to the TEM measurement, the samples were crushed and deposited on carbon coated Au grid after sonication of the powder into chloroform. Energy dispersive X-ray (EDX), dot maps, and selected area electron diffraction (SAED) have been also performed for obtaining the elemental analysis and the structural identification of the crystallites, respectively.

### 2.3. Catalytic Activity Tests

Measurements of the catalytic activity in CO total oxidation have been carried out for the three catalysts (10Ni–Al, 10Co–Al and 10Cu–Al) in the same reaction temperature range (from ambient temperature to 500 °C). The reaction was performed on the fresh catalysts placed in a fixed bed reactor, with an internal diameter of 16 mm, at atmospheric pressure. Only the 40–60 mesh particle size fractions of the sieved catalysts were used for the catalytic tests. The reactor was placed and heated, from ambient temperature to the desired reaction temperature, at a rate of 5 °C min^−1^ in an electric tubular oven. The reactor was fed with a gas reaction mixture with the following volumetric composition: 0.05%vol CO and 1.0%vol O_2_ in N_2_. The reaction temperature was continuously monitored by means of a thermocouple placed in the middle of the catalytic bed. The catalyst mass was chosen to have a space velocity (GHSV) value of ≈0.0376 g_CO_/(g_cat_ h) with a total gas flow rate of 50 NLh^−1^. The flow rate of CO and air in the reactor was regulated by digital gas flow meters.

The outlet gas products were quantitatively analyzed by an online gas analyzer. The conversion of CO was calculated using Equation (1):

(1) $$COconversion\;\%=(\left({\left[CO\right]}_{in}-{\left[CO\right]}_{out})/{\left[CO\right]}_{in}\right)\times 100,$$

where [*CO*]*_in_* and [*CO*]*_out_* represent the CO concentrations in the inlet gas and outlet gas, respectively.

## 3. Results and Discussion

### 3.1. Physico-Chemical Characterization

#### 3.1.1. XRD

**Figure 1:**
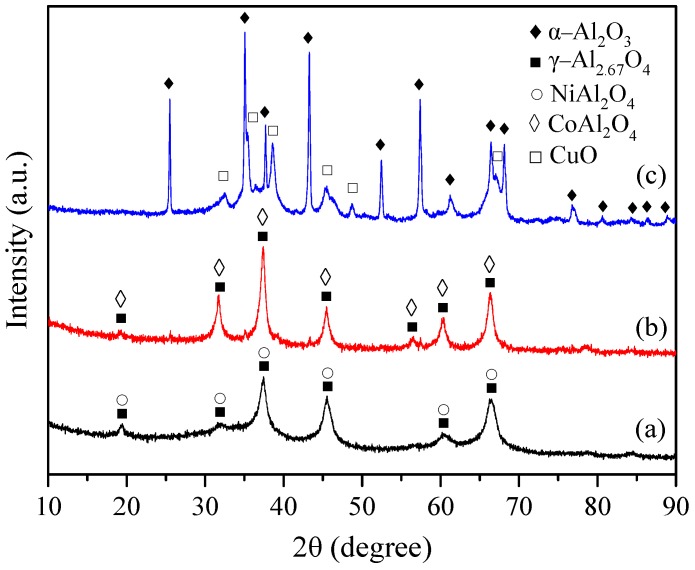
XRD patterns of (**a**) 10Ni–Al; (**b**) 10Co–Al and (**c**) 10Cu–Al calcined sample.

The XRD patterns of the different samples are shown in Figure 1. For the 10Ni–Al catalyst sample, the diffraction peaks at 2θ = 19.4°, 31.9°, 37.4°, 45.5°, 60.3° and 66.5° were attributed to NiAl_2_O_4_ (JCPDS 01-078-6951). The comparison of the slightly higher 2θ values of the formed spinel with those of a stoichiometric NiAl_2_O_4_ spinel provided by JCPDS file 01-078-6951 (2θ = 19.1°, 31.5°, 37.1°, 45.1°, 59.8° and 65,7°), indicates that a non-stoichiometric NiAl_2_O_4_ phase was formed [36]. The diffraction peaks at 19.4°, 31.9°, 37.4°, 45.5°, 60.3° and 66.5° can be assigned to a defect spinel of γ-alumina (γ-Al_2.67_O_4_) (JCPDS 00-047-1292), since its diffraction lines overlapped with those of nickel aluminate. For the 10Co–Al catalyst, the diffraction peaks at 2θ = 19.2°, 31.6°, 37.4°, 45.5°, 56.4°, 60.3° and 66.5° have been attributed to a defect spinel of γ-alumina (γ-Al_2.67_O_4_) (JCPDS 00-047-1292), which overlaps with the peaks of stoichiometric CoAl_2_O_4_ spinel (JCPDS 00-003-0896). Unfortunately, it is difficult to distinguish the diffraction peaks of Co_3_O_4_ because this phase presents overlapped diffraction lines with the γ-Al_2.67_O_4_ and CoAl_2_O_4_ phases. NiO and Co_3_O_4_ phases are not easy to be identified, suggesting that these species may be highly dispersed over the γ-Al_2.67_O_4_ surface, incorporated into the γ-Al_2.67_O_4_ lattice to form NiAl_2_O_4_ and CoAl_2_O_4_ spinels, or that the size of their crystallites was smaller than the detection limit of XRD measurements. The 10Cu–Al catalyst exhibited diffraction peaks of α-Al_2_O_3_ (JCPDS 00-042-1468) at 2θ = 25.5°, 35.1°, 43.3° and 57.4°, as well as diffraction peaks of CuO (JCPDS 00-005-0661) centered at 2θ = 32.5°, 35.5°, 38.5°, 45.3°, 48.8°, and 67°. Contrary to the other samples, no CuAl_2_O_4_ spinel phase was detected. The average crystallite size of CuO was 52 nm, as estimated by applying the Scherrer’s equation to the (111) plane at 2θ = 38.7°.

The XRD pattern obtained for the 10Ni–Al catalyst was quite similar to that reported in previous studies [37,38,39,40,41]. Li et al. [37] reported that a defect NiAl_2_O_4_ phase was formed on 10–13 wt % Ni–Al_2_O_3_ catalysts prepared by co-precipitation and impregnation. Kiš et al. [38] registered the formation of surface spinel, NiAl_2_O_4_, in co-precipitated 10 wt % NiO–Al_2_O_3_ catalyst. Zhao et al. [39] found that, for 10 wt % Ni–Al_2_O_3_ catalyst prepared by solution combustion method, only diffraction peaks of NiO and NiAl_2_O_4_ were present. Zangouei et al. [40] and Ewbank et al. [41] detected the presence of γ-Al_2_O_3_ and NiAl_2_O_4_ phases in 10 wt % Ni–Al_2_O_3_ catalysts prepared by the sol-gel method and by wet impregnation on solution combustion derived alumina. The authors suggested that the NiO species were well dispersed on the support by formation of NiAl_2_O_4_ phase. The XRD pattern of the 10Co–Al catalyst is in agreement with those reported in the literature [28,31]. In their study, Yao et al. [28] found that cobalt aluminate was formed during pretreatment of impregnated cobalt oxide on alumina at 600 °C. Wang et al. [31] found that Co_3_O_4_ species were present in a 5 wt % Co–Al_2_O_3_ catalyst prepared by wet impregnation. These species can transform into the CoAl_2_O_4_ phase by heat treatment above 700 °C. Richardson et al. [42] found that cobalt oxide-alumina systems, prepared by co-precipitation, present a mixture of Co_3_O_4_ and defect spinel Al_2_O_3_ (Al_8/3_Υ_1/3_O_4_, Υ: vacancy), which can be represented by the Co_m_Al_n_Υ_t_O_4_ spinel formula. The XRD pattern of 10Cu–Alwas different than those obtained for CuO–Al_2_O_3_ catalysts prepared by impregnation method [32]. The authors reported on the formation of CuO species and CuAl_2_O_4_. The formed CuAl_2_O_4_ spinel is the result of a solid–solid interaction between CuO and Al_2_O_3_ once the calcination temperature reaches 700 °C. In the most of the previously reported studies [28,31,32,37,38,39,40,41,42,43], it was observed that, in NiO–γ-Al_2_O_3_ and Co_3_O_4_–γ-Al_2_O_3_ systems, the metal oxide phase is present in two forms; a “bulk-like” metal oxide phase that segregates on the surface of the catalysts, and a dispersed phase present as stoichiometric and non-stoichiometric metal aluminates. This is due to the chemical interaction between the metal oxides and γ-Al_2_O_3_. The existence of this interaction appears to be a general characteristic of the most part of γ-Al_2_O_3_-supported/mixed oxide catalysts, since the γ-Al_2_O_3_ crystal structure corresponds to that of the spinel characterized by a deficit of cations. This interaction is expressed in terms of incorporation of metal ions into the alumina lattice sites of octahedral or tetrahedral symmetry. The incorporation of metal ions into tetrahedral sites (strong interaction) produces a metal “surface spinel”, which is probably similar to metal aluminate. Incorporation of metal ions into octahedral sites (weak interaction) leads to the formation of dispersed metal oxide species close to the surface. When the saturation of all available lattice sites is reached (monolayer coverage), the “bulk-like” metal oxide phases segregate on the surface of the catalyst. Indeed, a greater proportion of the metal ions diffuse into the lattice sites of alumina when the temperature increases, and eventually gives rise to the formation of a solid solution of bulk aluminate.

XRD studies suggested that a mixture of defect spinel γ-alumina (γ-Al_2.67_O_4_) and metal aluminate (MAl_2_O_4_) were present in the structure of the 10Ni–Al and 10Co–Al catalysts. On the other hand, CuO and α-Al_2_O_3_ separated phases were identified for the 10Cu–Al catalyst. The formation of MAl_2_O_4_ spinels in 10Ni–Al and 10Co-Al catalyst samples was correlated to the chemical interaction between the metal oxide and γ-alumina. The high flame temperature, reached during the combustion reaction, and the existence of spinel γ-alumina, with a deficit of cations, support this interpretation of the phenomenon.

#### 3.1.2. Adsorption/Desorption Isotherms

**Figure 2:**
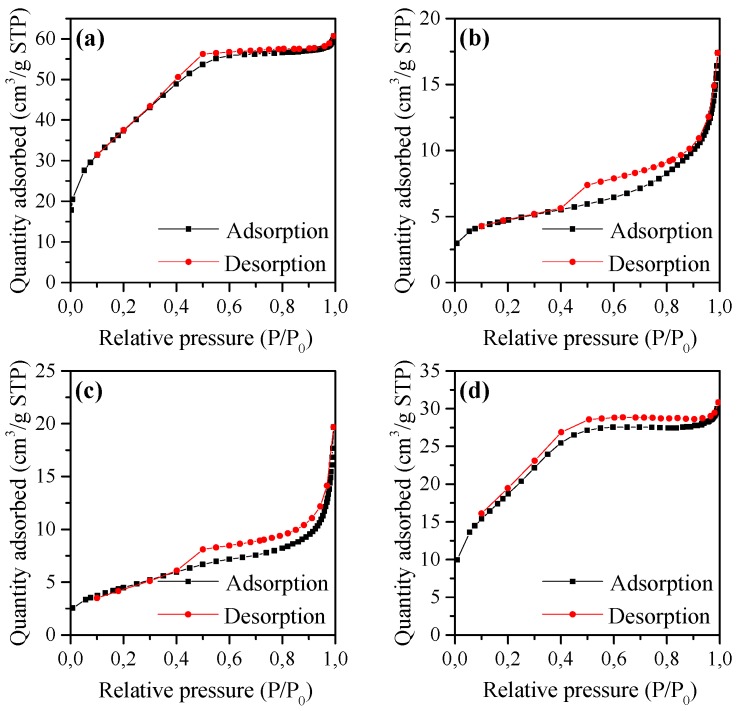
Nitrogen adsorption/desorption isotherms of (**a**) 10Ni–Al, (**b**) 10Co–Al (**c**) 10Cu–Al and (**d**) Al_2_O_3_ calcined sample.

Figure 2a–d shows the N_2_ adsorption/desorption isotherms of the alumina and mixed oxide samples. As revealed in Figure 2a,d, the isotherms of Al_2_O_3_ and 10Ni–Al samples are of type I, characteristic of microporous solids with relatively low external surface areas, with H4-type hysteresis loops that can be associated with the presence of narrow slit-like pores. In addition, 10Co–Al and 10Cu–Al samples showed type II isotherms (Figure 2b,c), characteristic of non-porous or macroporous solids, with H3-type hysteresis loops also related to aggregates of plate-like particles, giving rise to slit-shaped pores. The textural properties and the metal loadings (wt %) of the different calcined solids are summarized in Table 1. The surface area for the catalyst samples varied from 135.1–16.1 m^2^g^−1^ and the pore volume ranged between 0.024–0.084 cm^3^g^−1^, while the average pore size increased from 2.7–7.9 nm. It is worth noticing that the surface area values varied proportionally to the pore volume, while the average pore diameter increased with the diminution of the pore volume. The relatively low surface area, obtained for all samples, can be associated with the high flame temperature reached during the combustion process. Indeed, a high flame temperature leads to a slight sintering of the particles, resulting in a more extent agglomeration of the particles and consequently to low surface areas. The textural properties of the different samples are most probably linked to the formed metal-oxide phases. The highest surface area of 135.1 m^2^g^−1^ obtained for the 10Ni–Al sample could be associated with the presence of γ-alumina phase. The low surface area measured for the 10Co–Al sample is probably due to the formation of bulk cobalt aluminates, which are characterized by a low surface area [44]. For the 10Cu–Al catalyst, the presence of metal oxide particles seemed to block the α-Al_2_O_3_ pores, resulting in a slightly lower surface area when compared to that of the α-Al_2_O_3_ sample.

#### 3.1.3. XPS

**Figure 3:**
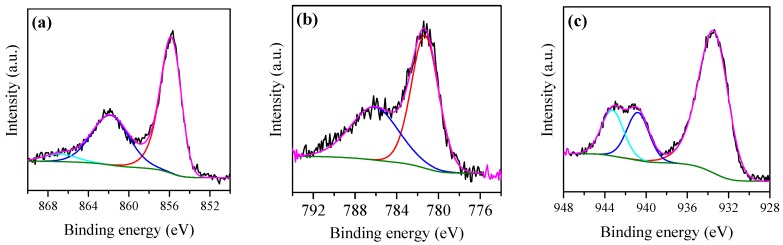
(**a**) Ni 2p_3/2_ XP spectra for 10Ni–Al calcined sample, (**b**) Co 2p_3/2_ XP spectra for 10Co–Al calcined sample and (**c**) Cu 2p_3/2_ XP spectra for 10Cu–Al calcined sample.

The XPS spectra of the calcined 10Ni–Al, 10Co–Al and 10Cu–Al samples are displayed in Figure 3a–c. The 10Ni–Alsample was characterized by a Ni 2p_3/2_ main peak centered at a binding energy (B.E.) of 855.8 eV, accompanied by two satellites at higher binding energies of 861.9 and 866.8 eV, corresponding to the presence of Ni^2+^ in NiAl_2_O_4_ [35]. The presence of Ni^2+^ in NiO was clearly excluded because the characteristic band at 853.7 eV was not present [45,46,47,48]. The band at 855.8 eV could also be assigned to Ni^2+^ in Ni(OH)_2_, but the absence of asymmetry for the peak at 861 eV excluded the presence of Ni(OH)_2_ [47]. The Co 2p_3/2_ spectrum, acquired for the 10Co–Al catalyst, presented a peak at 781.29 eV and its satellite at around 786.13 eV. The binding energy value of Co 2p_3/2_ was close to that of Co^2+^ in CoAl_2_O_4_ reported by Duan et al. [49] (781.68 eV) and Han et al. [50] (781.34 eV), indicating the presence of CoAl_2_O_4_ on the sample surface. The absence of peaks around 780 eV indicates that Co_2_O_3_, CoO and Co_3_O_4_ species are absent [45]. Moreover, no peaks were detected around 780.4 eV, indicating the absence of the Co(OH)_2_ phase [45]. The 10Cu–Al sample presented a Cu 2p_2/3_ peak centered at 933.5 eV and two satellite peaks at B.E. of 940.79 and 943.26 eV, corresponding to Cu^2+^ in CuO [51,52,53]. The use of the modified Auger parameter (Cu2p_3/2_, Cu LMM) (1851.5 eV) accurately confirmed the existence of CuO, in agreement with the data previously reported in the literature [54]. In addition, no CuAlO_2_ and Cu_2_O phases were present in the sample due to the absence of peaks at around 932 eV and 932.4 eV, respectively [54,55,56]. In the same way, the absence of peaks around 935 eV, confirmed the absence of Cu(OH)_2_ and CuAl_2_O_4_ phases [35,51,52,54].

#### 3.1.4. SEM

**Figure 4:**
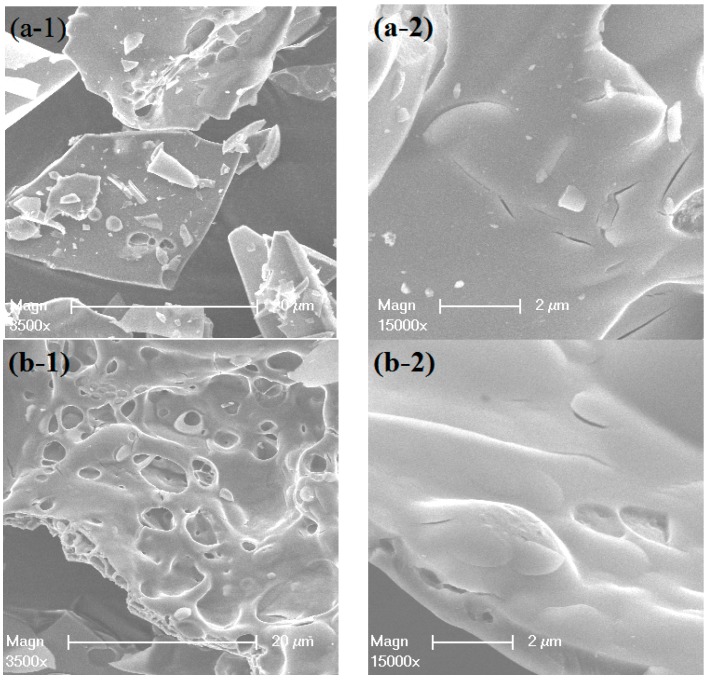

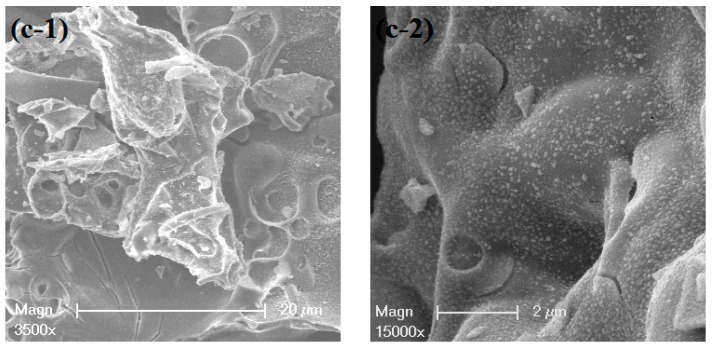
SEM images of (**a**) 10Ni–Al, (**b**) 10Co–Al and (**c**) 10Cu–Al calcined samples.

Figure 4 shows the SEM micrographs of the agglomerates of the samples. The morphology and the surface features of the various calcined samples are different. The 10Ni–Al catalyst presented a plate and “flaky-like” morphology with an average size of the agglomerates higher than 50 µm. A careful analysis of the 10Ni–Alsample micrograph revealed that the agglomerates were constituted by nearly hexagonal platelet particles (Figure 4(a-1)). The 10Co–Al and Cu–Al samples were characterized by foamy and flaky like particles with an average size higher than that of 10Ni–Al. Figure 4(b-1,c-1) show the presence of voids with irregular shapes in the alumina matrix, which were attributed to the high volume combustion gases released during the combustion process. The observed foamy morphology might facilitate the access to the internal surface of the catalyst and potentially improve the catalytic activity. The particles of the 10Ni–Al and 10Co-Alsamples presented smoothed surfaces (Figure 4(a-2,b-2)), while the surface of 10Cu–Alappeared to be homogenously covered by quasi-spherical nanoparticles (Figure 4(c-2)), which could be attributed to the CuO particles.

#### 3.1.5. TEM-EDX and Elements Mapping

**Figure 5:**
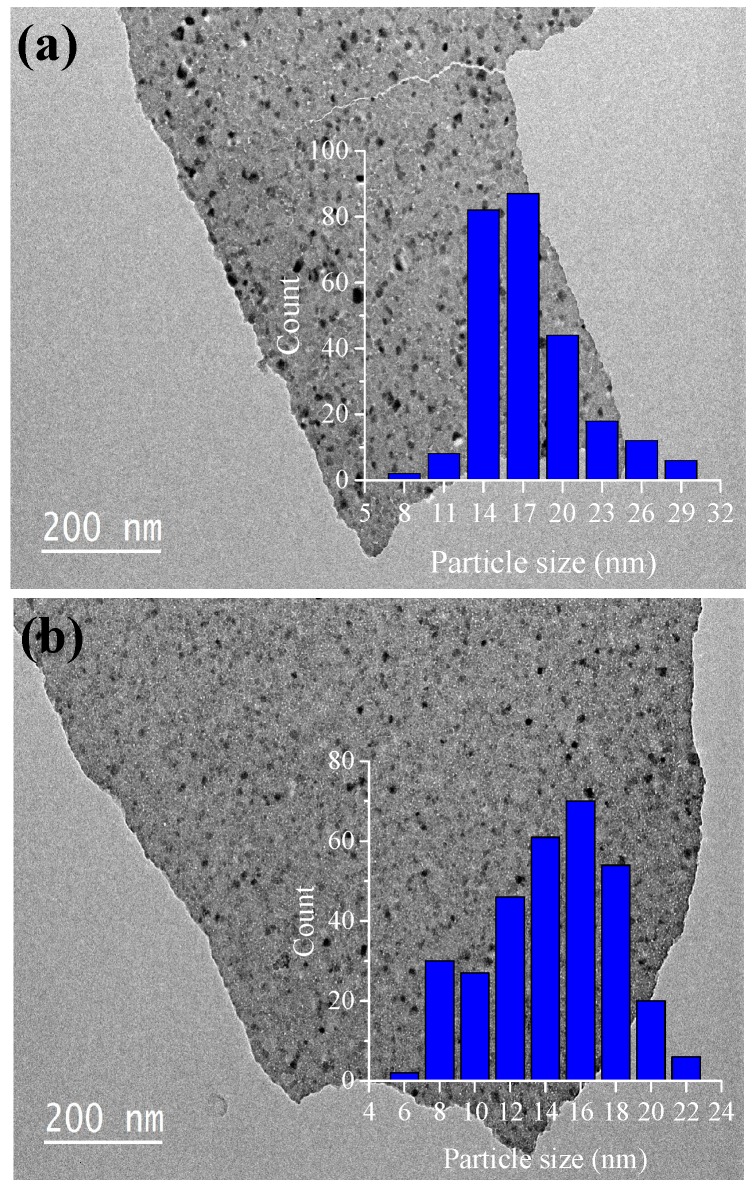

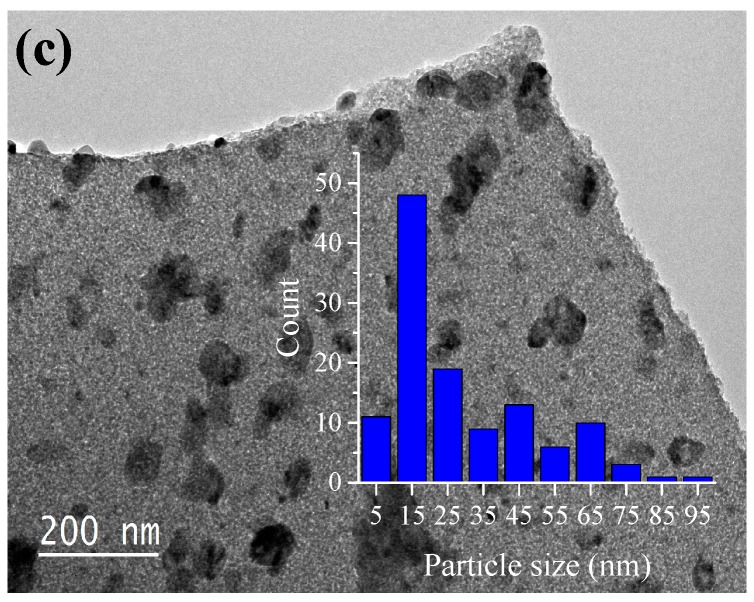
TEM images (inset, nanoparticle size distribution) of (**a**) 10Ni–Al, (**b**) 10Co–Al and (**c**) 10Cu–Al calcined samples.

The TEM micrographs of the different fresh catalyst samples are displayed in Figure 5a–c. TEM images show the presence of Cu, Co, or Ni metal oxide nanoparticles on the surface of the alumina matrix. The metal oxide nanoparticles, corresponding to dark spots, could be clearly distinguished from the light grey alumina phase. In Figure 5a,b, for the 10Ni–Al and 10Co–Al samples, the metal oxide nanoparticles appeared to be uniformly dispersed on the entire surface of the bulk Al_2_O_3_ phase. No agglomeration phenomenon was observed. Differently, for the 10Cu–Alsample, an irregular distribution and agglomeration of copper oxide crystallites were observed in Figure 5c. The histograms of nanoparticle size distribution are shown in the inset of Figure 5a–c. 10Ni–Al and 10Co–Alsamples presented smaller particle size and narrower distribution of nanoparticles than the 10Cu–Al catalyst. Furthermore, 10Ni–Al and 10Co–Al samples showed particles in the range of 8–29 nm and 6–22 nm, respectively, while the 10Cu–Al catalyst presented a much larger distribution (5–95 nm) with the highest population centered at 15 nm. Although TEM observations were not conclusive, they suggested that the Ni and Co-containing phases were dispersed on the alumina surface as CoAl_2_O_4_ and NiAl_2_O_4_ “surface spinels”. For the 10Cu–Al catalyst, copper was mainly present as CuO. Because of the low surface area of 10Cu–Al, the dispersion of the metal oxide particles was less efficient, and CuO nanoparticles partially aggregate on the alumina surface, thus resulting in the increasing of the particle sizes. These first observations were then deeply studied by performing EDX mapping and SAED-TEM.

**Figure 6:**
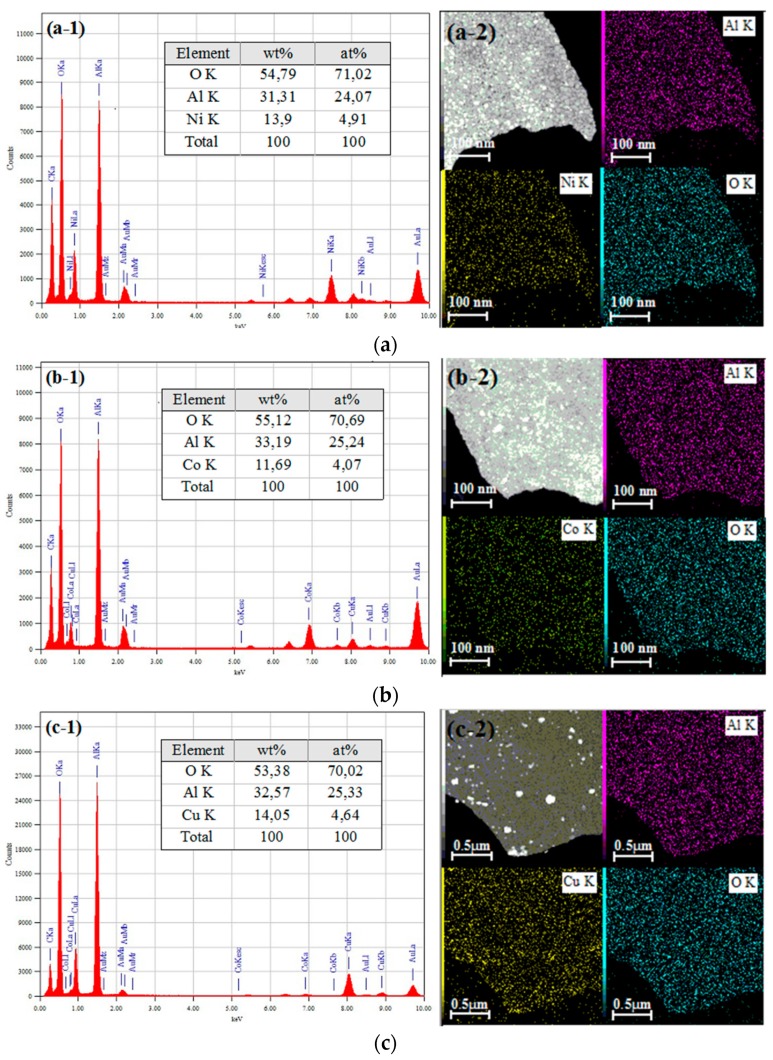
TEM-EDX Dot mapping analyses of (**a**) 10Ni–Al, (**b**) 10Co–Al and (**c**) 10Cu–Al calcined samples.

TEM-EDX and element mapping analyses of the calcined samples are reported in Figure 6. The EDX spectra (Figure 6(a-1–c-1)) confirmed that all the samples did not contain unexpected impurities. The table inserted in Figure 6(a-1–c-1) reports the element content of the catalysts. The nickel, cobalt and copper content in the 10Ni–Al, 10Co–Al, and 10Cu–Al samples was higher than that theoretically expected from the amount of precursors added during the preparation of the samples (10 wt %), and from that measured by X-ray fluorescence. The main difference between XRF and EDX results can be explained by the different probing depth provided by these two techniques. Indeed, TEM-EDX probe the material up to 100 nm depth, thus giving information only of the composition of the surface layer of the samples. As visualized in Figure 6(a-2,b-2), Ni and Co elements were highly dispersed on the surface of 10Ni–Al and 10Co–Al solids. In contrast, Figure 6(c-2) shows a nonhomogeneous distribution of Cu-containing species on the surface of 10Cu–Alparticles. These results are consistent with the observation obtained by the observation of TEM micrographs that showed that “bulk-like” CuO phase aggregates on the surface of 10Cu–Al lead to a high local Cu concentration. On the other hand, Ni and Co elements can be highly dispersed on the alumina surface if they are incorporated into the spinel structure of alumina to form metal aluminate (MAl_2_O_4_). In this case, rather than being completely incorporated into the alumina structure, a surface spinel may be formed.

**Figure 7:**
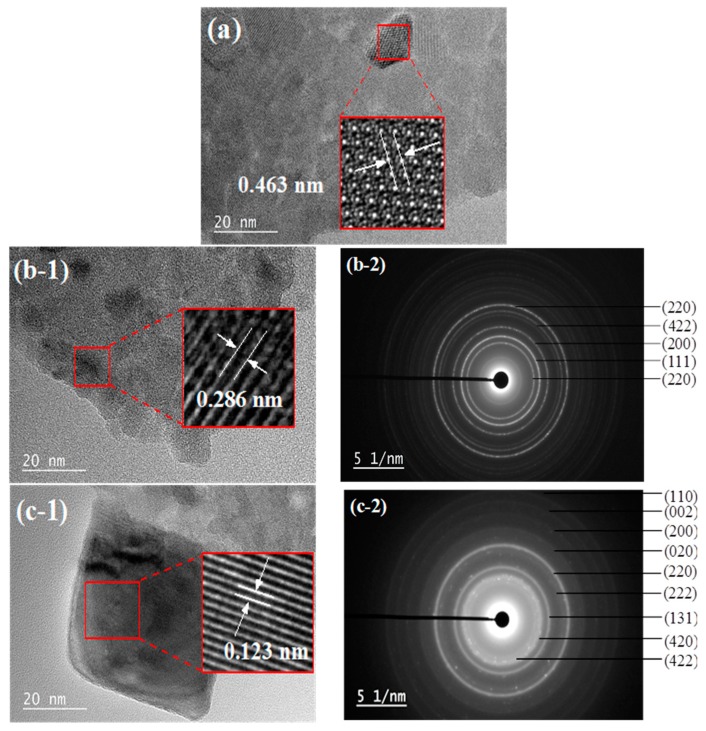
Lattice fringes and Selected Area Electron Diffraction (SAED) patterns of (**a**) 10Ni–Al, (**b**) 10Co–Al and (**c**) 10Cu–Al calcined samples.

In order to obtain more detailed information on the structure of the crystallites, the samples have been characterized by TEM-Selected Area Electron Diffraction (SAED). TEM images of the samples and their corresponding SAED patterns are shown in Figure 7. According to Figure 7a, the d-spacing of the lattice fringe of 10Ni–Al sample was of 0.463 nm, corresponding to the cubic NiAl_2_O_4_ (111) plane. Figure 7b-1 shows a fringe spacing of 0.286 nm, corresponding to the cubic CoAl_2_O_4_ (220) plane. The SAED pattern of the region highlighted in the red square in Figure 7(b-1) is reported in Figure 7(b-2). The SAED patterns of the 10Co–Al sample consisted of continuous concentric rings that could be assigned to CoO (220), (111), (200), and to CoAl_2_O_4_ (422), (220). The SAED results confirmed the presence of NiAl_2_O_4_ and CoAl_2_O_4_ “surface spinels” in the 10Ni–Al and 10Co–Al samples, respectively. For the 10Cu–Al sample, a lattice distance of 0.123 nm was observed and assigned to the Cu_2_O (222) lattice spacing (Figure 7(c-1)). In the corresponding SAED patterns, the discrete diffraction spots formed discontinuous concentric rings corresponding to monoclinic CuO (110), (002), (020), (131), (420), and cubic Cu_2_O (200), (220), (222), (422) (Figure 7(c-2)). The formation of non-incorporated CuO particles on the alumina surface, which can be a key factor to ensure a good catalytic performance, was also confirmed. These observations are consistent with the XPS results that showed that the nickel and cobalt phases are dispersed on the alumina matrix as “surface spinels”.

The surface and the bulk proprieties suggested that a mixture of γ-alumina and NiAl_2_O_4_ “surface spinel”/bulk phases were present in the 10Ni–Al sample. For the 10Co–Al sample, a mixture of γ-alumina and CoAl_2_O_4_ “surface spinel”/bulk phases were observed accompanied by a low amount of CoO species. Contrary to the 10Ni–Al and 10Co–Al samples, a mixture of α-Al_2_O_3_ and “bulk-like” CuO separated phases, as well as small amount of Cu_2_O species, were present on the 10Cu–Al sample.

### 3.2. Evaluation of the Catalytic Activity of the Catalysts in CO Total Oxidation

**Figure 8:**
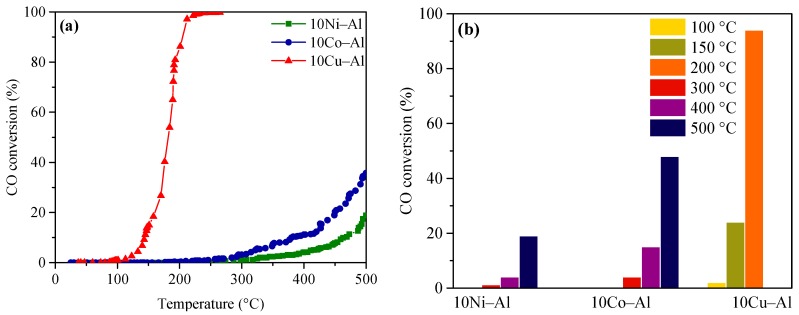
(**a**) CO conversion and (**b**) effect of reaction temperature on CO conversion over the calcined catalysts.

The conversion of CO on the 10Ni–Al, 10Co–Alor 10Cu–Al catalysts, as a function of the reaction temperature (up to 500 °C), is presented in Figure 8a. The catalysts showed a clear difference in their CO oxidation activity. In fact, for the 10Cu–Alsample, the CO conversion began at low temperature (around 80 °C) and rapidly increased with the temperature in the 80–250 °C range, reaching 100% conversion. For the two other catalysts, the conversion of CO started at higher temperatures (around 250 °C) and gradually increased with the temperature, but never reached the total conversion of CO. A comparison of the light-off temperature for all the catalysts is reported in Table 2. T_1_, T_50_ and T_100_ respectively represent the characteristic temperatures for the start-up of the oxidation, the half, and the full conversion of CO into CO_2_. As reported in Table 2, the oxidation of CO began at 301, 246, and 97 °C over the 10Ni–Al, 10Co–Al and 10Cu–Al catalysts, respectively. The temperature, at which 50% conversion was reached (T_50_), was equal to 497 °C and 180 °C, for 10Co–Al and 10Cu–Al, respectively. The maximum CO conversion achieved at 500 °C over the 10Ni–Al and 10Co–Al samples was of 19% and 52%, respectively. Total CO conversion was obtained only for the 10Cu–Al catalyst already at 250 °C.

To further check the influence of the reaction temperature on the CO conversion, the activity of the catalysts was measured at three different reaction temperatures, during 30 min on-stream, in the 100–500 °C range, as shown in Figure 8b. The 10Cu–Al catalyst exhibited a very low CO conversion (less than 5%) at 100 °C. Then, the reaction ran-out to reach a CO conversion of 87% at 200 °C. The 10Co–Al catalyst exhibited low CO conversion values even at relatively high temperature (around 4% and 15% at 300 °C and 400 °C, respectively). The 10Ni–Al catalyst presented the lowest performances, with a CO conversion lower than 5% at 300 °C and 400 °C. At a higher reaction temperature (500 °C), 10Co–Al significantly outperformed the 10Ni–Al catalyst; the CO conversion measured for 10Co–Al was the double of that of the 10Ni–Al sample. The catalytic activity was strongly affected by the reaction temperature probably due to the influence of the surface redox reactions. At low temperature, CO can adsorb on the surface of the metal oxide and saturate all active sites, thus inhibiting the adsorption of molecular O_2_. By increasing the temperature (up to around 300 °C), CO starts to desorb from the surface of the catalysts, leaving free sites on which O_2_ can adsorb. The conversion of CO can then take part. A further increase of the temperature provokes a further CO desorption, thus resulting in an improved conversion of CO.

To conclude, the 10Cu–Al catalyst showed the highest CO oxidation activity at a low reaction temperature when compared to the other samples. The order of activity of the different catalysts was the following: 10Cu–Al > 10Co–Al > 10Ni–Al. The difference in catalytic activity is probably related to their different surface properties. The highest CO oxidation activity observed for 10Cu–Al is due to the absence of “surface spinel” phase, which is known to be inactive in CO oxidation [32,33,34]. The 10Cu–Al catalyst better performed than similar catalysts previously studied in the literature. As an example, Pierron et al. [33] and Luo et al. [34] obtained, for two 10Cu–Al catalysts prepared by wet impregnation, a CO conversion of 50% at around 250 °C and 270 °C, while the 10Cu–Al catalyst prepared in the present work showed complete CO conversion at the same temperature. On the other hand, the low activity of the Ni and Co-containing catalysts can be attributed to the formation of low active CoAl_2_O_4_ and NiAl_2_O_4_ “surface spinels”. The CO oxidation activity observed for 10Ni–Al is quite similar to that reported by Chen et al. [35]. However, a much better CO oxidation activity at lower temperature was observed for 10Co–Alin previous studies [28,29,30,31]. In most cases, cobalt oxide gives an interesting activity for CO oxidation, whereas its activity dramatically decreased when Co is present as CoAl_2_O_4_.

## 4. Conclusions

Alumina-based Ni, Cu, Co-oxide catalysts prepared through microwave-assisted solution combustion were fully characterized and tested in CO oxidation. Correlation between the catalyst surface properties and the catalytic performances could be established. The results showed that, for the 10Ni–Al and 10Co–Alcatalysts, Ni and Co were mainly present as metal-aluminate surface spinels that are very low active species in CO oxidation. On the other hand, the 10Cu–Alcatalyst showed well defined α-Al_2_O_3_ and CuO phases nanometrically dispersed, and very good catalytic performances.

Microwave-assisted solution combustion reveals itself to be a promising method to synthesize very active catalysts: 10Cu–Al catalyst showed a very high activity in CO oxidation at low temperature, much higher than that reported in the literature for similar catalysts. Nevertheless, further investigations have to be done in order to optimize the synthesis parameters and minimize the spinel formation for Ni–Al and Co–Al binary oxide catalysts.

## Figures and Tables

**Table 1:** Physico-chemical of the calcined samples.

Catalyst	Metal Loading ^1^ (wt %)	BET Surface (m^2^g^−1^)	Pore Volume ^2^ (cm^3^g^−1^)	Pore Diameter ^3^ (nm)
α-Al_2_O_3_	-	69.2	0.044	2.6
10Ni–Al	10.3	135.1	0.084	2.7
10Co–Al	10.7	16.1	0.024	7.9
10Cu–Al	10.7	16.4	0.031	6.5

^1^ XRF elemental analysis. ^2^ BJH Desorption average pore diameter. ^3^ BJH Desorption pore volume.

**Table 2:** The light-off temperature of catalyst samples for CO oxidation.

Catalyst	T_1_	T_50_	T_100_
10Ni–Al	301	–	–
10Co–Al	246	497	–
10Cu–Al	97	180	250
